# Exclusive Breastfeeding Rate and Complementary Feeding Indicators in China: A National Representative Survey in 2013

**DOI:** 10.3390/nu10020249

**Published:** 2018-02-22

**Authors:** Yifan Duan, Zhenyu Yang, Jianqiang Lai, Dongmei Yu, Suying Chang, Xuehong Pang, Shan Jiang, Huanmei Zhang, Ye Bi, Jie Wang, Robert W. Scherpbier, Liyun Zhao, Shian Yin

**Affiliations:** 1 Key Laboratory of Trace Element Nutrition of National Health and Family Planning Commission, National Institute for Nutrition and Health, Chinese Center for Disease Control and Prevention, No. 29 Nanwei Road, Xicheng District, Beijing 100050, China; duanyf@ninh.chinacdc.cn (Y.D.); yangzy@ninh.chinacdc.cn (Z.Y.); yudm@ninh.chinacdc.cn (D.Y.); pangxh@ninh.chinacdc.cn (X.P.); jiangshan@ninh.chinacdc.cn (S.J.); zhanghm@ninh.chinacdc.cn (H.Z.); biye@ninh.chinacdc.cn (Y.B.); wangjie@ninh.chinacdc.cn (J.W.); zhaoly@ninh.chinacdc.cn (L.Z.); shianyin@126.com (S.Y.); 2 United Nations Children’s Fund-China Office, No. 12 Sanlitun Road, Chaoyang District, Beijing 100600, China; schang@unicef.org (S.C.); rscherpbier@unicef.org (R.W.S.)

**Keywords:** cross-sectional study, infant and young child, breastfeeding, complementary feeding

## Abstract

Appropriate infant and young child feeding could reduce morbidity and mortality and could improve cognitive development of children. However, nationwide data on exclusive breastfeeding and complementary feeding status in China are scarce. The aim of this study was to assess current exclusive breastfeeding and complementary feeding status in China. A national representative survey (Chinese National Nutrition and Health Survey) of children aged under 6 years was done in 2013. Stratified multistage cluster sampling was used to select study participants. World Health Organization (WHO) infant and young child feeding indicators were firstly used to assess exclusive breastfeeding and complementary feeding practice nationwide. In total, 14,458 children aged under two years (0 to <730 days) were studied from 55 counties in 30 provinces in China. The crude exclusive breastfeeding rate under 6 months was 20.7% (908/4381) and the weighted exclusive breastfeeding rate was 18.6%. The crude prevalence of minimum dietary diversity, minimum meal frequency and minimum acceptable diet were 52.5% (5286/10,071), 69.8% (7027/10,071), and 27.4% (2764/10,071) among children aged 6–23 months, respectively. The weighted rate was 53.7%, 69.1%, and 25.1%, respectively. Residential area, household income and maternal education were positively associated with the three complementary feeding indicators. The exclusive breastfeeding rate under 6 months was low and complementary feeding practice was not optimal in China. Residential area, household income and maternal education might be used to target infants and young children to improve complementary feeding practice.

## 1. Introduction

The first 1000 days (from conception to a child’s second birthday) is a critical period for good human nutrition, health and development, of which the benefit could last throughout the whole life [1]. Malnutrition (undernutrition, micronutrient deficiency and overweight/obesity) during this period could increase the risk of morbidity and mortality [2]. It was estimated that undernutrition caused 45% of child deaths under five years, which were 3.1 million child deaths worldwide annually [2]. In the long run, nutrition during the first 1000 days may have an impact on adulthood health.

Appropriate infant and young child feeding is a prerequisite for optimal child nutrition, health and development [3]. The World Health Organization (WHO) recommends that infants should be exclusively breastfed for the first six months and complementary foods should be introduced after that with continued breastfeeding until two years or beyond [4]. Breastfeeding could protect child against infectious diseases and malocclusion and increase intelligent quotient [5]. Suboptimal breastfeeding could cause 804,000 child deaths or 11.6% of total child deaths globally [2]. Growth faltering becomes the most evident during the complementary feeding period [6]. Micronutrient deficiency is highly prevalent during the period too [7]. Although the prevalence of malnutrition decreased dramatically for children under five years during the past two decades in China, it remains high during the complementary feeding period [8].

Current indicators used to assess both breastfeeding and complementary feeding were firstly introduced by WHO, United Nations Children’s Fund (UNICEF) and other related institutes in 2008 [9], and the measurement questionnaire of these indicators was published in 2010 [10]. We adopted these indicators and related measurement tools in the Chinese National Nutrition and Health Survey (CNNHS) in 2013 to assess the infant and young child feeding status in China. Four core indicators were reported here and the associated factors were explored in this study, including exclusive breastfeeding (EBF), minimum dietary diversity (MDD), minimum meal frequency (MMF) and minimum acceptable diet (MAD).

## 2. Materials and Methods

### 2.1. Subjects

Children under 2 years of age were categorized into 3 age groups (0–5 months, 6–11 months and 12–23 months). In each age group, 90 children were randomly sampled in each selected county, which was described in detail below. Their caregivers were asked to finish a face-to-face interview. The Ethics Review Board of the National Institute for Nutrition and Health, Chinese Center for Disease Control and Prevention approved the protocol (2013–018). All caregivers gave their informed consent in writing to participate before starting the interview.

### 2.2. Study Design

The study was a part of CNNHS in 2013. The detailed methods were described previously [11]. Briefly, this was a cross-sectional survey among children under 6 years of age and lactating mothers from 30 provinces, autonomous regions and municipalities in mainland China (Tibet autonomous region was not included in the survey). Multi-stage stratified cluster random sampling was used in the study. In total, 2865 districts/counties in China were categorized into 4 strata (large cities, medium and small cities, general rural areas and poor rural areas), of which 55 counties (12 metropolis, 15 medium and small cities, 18 general rural areas, and 10 poor rural areas) were chosen in the study. In each selected county, 3 communities/townships were systematically sampled. In each selected township, 3 neighborhood/villages were systematically selected. Finally, 10 children from each age group were randomly selected in each selected village. The total sample size was 34,650 for children under 6 years, of which 14,850 was for children under 2 years. Sample size calculation was based on estimation of the prevalence of anemia for children under 6 years with taking complex sampling design into account.

### 2.3. Study Outcomes

A standard questionnaire was adapted from WHO/UNICEF indicators for assessing infant and young child feeding practice and measurement [10]. Exclusive breastfeeding under 6 months, minimum dietary diversity, minimum meal frequency and minimum acceptable diet were included in the article, which were firstly assessed in a national survey in China. Exclusive breastfeeding was defined, as breast milk is the only food and liquid consumed with the exception of ORS (oral rehydration salt), drops and syrups (vitamins, minerals and medicines) for infants under 6 months. The exclusive breastfeeding rate under 6 months was calculated using the number of infants aged 0–5 months who are fed exclusively with breast milk during the previous day divided by the total number of living infants aged 0–5 months surveyed. Minimum dietary diversity was calculated using the number children 6–23 months of age who receive foods from 4 or more food groups of the 7 food groups (1. grains, roots and tubers; 2. legumes and nuts; 3. dairy products (milk, yogurt, cheese); 4. flesh foods (meat, fish, poultry and liver/organ meats); 5. eggs; 6. vitamin-A rich fruits and vegetables; and 7. other fruits and vegetables) during the previous day divided by the total number of children aged 6–23 months surveyed. Minimum meal frequency was calculated using the number of children aged 6–23 months who receive the minimum number of times or more solid, semi-solid, or soft foods (but also including milk feeds for non-breastfed children) during the previous day divided by the total number of children aged 6–23 months surveyed. Minimum acceptable diet was calculated using the number of children aged 6–23 months who received a minimum acceptable diet (apart from breast milk) during the previous day divided by the total number of children aged 6–23 months surveyed.

### 2.4. Statistical Analysis

All the data were analyzed using SAS 9.4 release (SAS Institute Inc., Cary, NC, USA). Data were entered via a standardized data management platform and were cleaned for all variables. Categorical variables were expressed as percentage (%). Complex survey sampling methods were taken into account in the analyses and sampling weight was applied to exclusive breastfeeding rate and complementary feeding indicators analyses. A Rao–Scott chi-square test was used for categorical variable comparison in the surveyfreq procedure. The surveylogistic procedure was used to assess the relationship between each indicator and the possible predictors (e.g., gender, ethnicity, residential areas, maternal age, maternal migrant status, maternal education, and annual household income level). Firstly, the bivariate analysis (surveyfreq) was conducted between each feeding indicator and each predictor. The variables marginally associated with outcome variables in the bivariate analysis were selected for multivariate analyses (*p* < 0.20). Then, multivariate analysis (surveylogistic) was conducted for each feeding indicator with potential risk factors. In the final model, only variables significantly associated with outcome variables were retained (*p* < 0.05). The adjusted odds ratio (OR) and 95% confidence intervals (CIs) were reported in the final logistic regression model while retaining all significant variables.

## 3. Results

In total, 14,458 infants and young children under two years old were surveyed in the study. The general characteristics were described in Table 1. The crude rate of exclusive breastfeeding under 6 months was 20.7% and the weighted rate was 18.6%. The crude rate of minimum dietary diversity, minimum meal frequency and minimum acceptable diet was 52.5%, 69.8% and 27.4% for 6–23 months infants and young children, respectively. The corresponding weighted rate was 53.7%, 69.1%, and 25.1%.

The weighted EBF rate was not significantly associated with either gender, early initiation of breastfeeding, residential area, ethnicity, maternal factors (e.g., age, educational level, migrant status) or annual household income level (Table 2). Only early initiation of breastfeeding was marginally related to EBF rate (OR: 1.27, 95% CI: 0.94–1.72).

Minimum dietary diversity was significantly associated with residential areas, maternal age group, maternal education, maternal migrant status and annual household income level (Table 3). Compared with children living in poor rural areas, the adjusted odds ratio for minimum dietary diversity was 2.46, 2.33 and 1.05 for those living in metropolis, medium or small cities, and general rural areas, respectively. Children whose mothers were younger than 24 years old had 27% less odds of consuming diverse diet than those children whose mothers were older than 30 years old. Children of migrant mothers had 1.62 times odds of consuming diverse diet than those children of mothers living at home. Children had 39% less odds of consuming diverse diet of maternal education equal to or less than nine years than those of maternal education more than nine years. Children from household with higher income (10,000–19,999 Chinese Yuan (CNY) and ≥20,000 CNY) had 1.65 and 1.63 times odds of consuming diverse diet than those from household with annual income less than 10,000 CNY per capita.

Minimum meal frequency was significantly associated with the residential area and maternal education level, but not associated with ethnicity, maternal age group, and annual household income level (Table 4). Compared with children living in poor rural areas, the odds ratio for minimum meal frequency was 3.52, 4.23 and 2.27 for those living in metropolis, medium or small cities, and general rural areas, respectively. Children of maternal education equal to or less than nine years had 27% less odds of meeting the minimum meal frequency than those with maternal education more than nine years.

Minimum acceptable diet was significantly associated with residential area, maternal age group, maternal education, and household income level (Table 5). Compared with children living in poor rural areas, the odds ratio for minimum acceptable diet was 4.59, 4.43 and 1.78 for those living in metropolis, medium or small cities, and general rural areas, respectively. Children whose mothers were younger than 24 years old had 30% less odds of consuming acceptable diet than those whose mothers were older than 30 years old. Children of maternal education equal to or less than nine years had 36% less odds of receiving a minimum acceptable diet than those of maternal education more than nine years. The household with annual per capita income greater than 20,000 CNY had greater odds of minimum acceptable diet than those household with annual per capita income less than 10,000 CNY (OR: 1.70, 95% CI: 1.30–2.23).

## 4. Discussion

This study firstly used the updated WHO exclusive breastfeeding and complementary feeding indicators to explore the possibility of improving infant and young child feeding status nationwide in mainland China. The exclusively breastfed rate was astonishingly low in China. Dietary diversity, meal frequency and acceptable diet during complementary feeding period were not optimal for 6–23 months infants and young children in China. Residential areas, maternal education level or household income were potential risk factors for suboptimal complementary food diversity and frequency. These characteristics could be used for targeting infants and young children to improve breastfeeding and complementary feeding practices in China.

The exclusive breastfeeding rate under 6 months was the lowest since 1950s in China. In the 1950s–1960s, breastfeeding under 6 months was very popular in China. About 90% of infants in the rural areas and 80% of infants in the urban areas were solely breastfed in Beijing [12]. In early 1980s, the predominantly breastfed rate went down dramatically and only about half of infants in the urban areas were predominantly breastfed under 6 months in China [13]. In 2008, the exclusive breastfeeding rate under 6 months was 28% in China [14]. Different measurements of exclusive breastfeeding may partially explain the difference. However, the rate in our study was even lower than the one defined with similar measurement in 2008. Recent data showed that exclusive breastfeeding rate under 6 months was about 40% globally and only 37% of infants under 6 months were exclusively breastfed in low-income or middle-income countries [5]. The EBF rate in China was on a downward tendency and obviously lower than the global average level.

Meanwhile, the time trend of exclusively breastfed rate from 1993 to 2013 increased by about 0.5 percentage point annually in low-middle income countries [5]. By contrast, the exclusive breastfeeding rate decreased by more than one percentage point annually from 2008 to 2013 in China. It is urgent to take action to control this down trend. As one of the six global nutrition targets set by World Health Assembly, the exclusive breastfeeding rate should reach at least 50% by 2025 [15]. There are enormous challenges to achieve this target in the next 10 years or so in China, which could have a great impact on achieving the global target. Therefore, more resources from both domestic and international societies are required to promote breastfeeding in order to dramatically increase the exclusive breastfeeding rate in China. The exclusive breastfeeding rate did not differ in either rural areas or urban areas, which requires that breastfeeding promotion should cover the whole country and none should be left behind. Early initiation of breastfeeding was only marginally associated with the exclusive breastfeeding rate.

Complementary feeding is continuously critical for nutrition and health of humans during their lifetimes. In the complementary feeding period, children are vulnerable to suffering from undernutrition. A few studies reported that diverse complementary foods were associated with high height-for-age *z*-scores [16]. Improving complementary food quality could increase either height or height-for-age *z*-score. Child malnutrition remains highly prevalent in low-income and middle-income countries, which could be related to the inappropriate complementary feeding practice. In a five-country study (Bangladesh, India, Nepal, Pakistan and Sri Lanka), minimum dietary diversity was low as 15% in India and high as 71% in Sri Lanka [17]. Only about one-tenth of children received a minimum acceptable diet and meal frequency met the recommendation for ~50% of children in Nigeria [18]. The minimum acceptable diet rates were quite low from 19.9% to 5.5% in West African countries [19]. In this study, the complementary feeding practice in China was also not optimal and the rates were alarmingly low, which deserves close attention for both public health workers and policy makers. Less than thirty percent of the children aged 6–23 months received a minimum acceptable diet, which was slightly better than those in South Asian and West African countries. Our data showed that older infants and young children living in metropolises, medium or small cities and general rural areas received more diverse complementary foods than those living in poor rural areas, even after being controlled for household income and maternal education level. In poor rural areas of China, the rates of complementary feeding indicators were consistently low and the prevalence of stunting was high. This implies that food availability, purchasing power, knowledge about complementary feeding might play some roles in different residential areas. Improving complementary feeding practice in poor areas could be integrated into the current national poverty alleviation action.

Maternal education and household income were potential determinants for suboptimal complementary feeding indicators. Both lower maternal education and lower household income were associated with lower complementary feeding practice consistently across the five Asian countries (Bangladesh, India, Nepal, Pakistan and Sri Lanka) [17]. Another study in Indonesia also showed that poorer household income or lower maternal education was associated with poor minimum dietary diversity [20]. In Ghana, poverty and maternal education were also risk factors for complementary feeding practice [21]. Nigerian children received more acceptable complementary foods in higher socioeconomic status group than their counterparts [18]. In the present study, maternal education and household income were also positively associated with dietary diversity and adequate diet. Children with younger mothers received less diverse complementary foods than older mothers. A study in Pakistan also supported that older mothers tended to give more diverse complementary foods than younger mothers [22].

Improving breastfeeding and complementary feeding practices needs to be coordinative rather than contradictive. The Chinese National Nutrition Plan (2017–2030) issued recently set the target for improving breastfeeding and reducing undernutrition for infants and young children, which brings a unique opportunity to coordinate breastfeeding promotion and complementary feeding improvement programs. Both breastfeeding and complementary feeding should be emphasized continuously through the first two years of life in China.

Despite a national representative survey with a large sample size, there are several limitations of this study. First, this is a cross-sectional study, which could not be used for establishing causal relationship between infant and young child feeding indicators and associated factors. However, these factors could be used to identify populations with priority for intervention. Second, there were some missing values (e.g., household income), but less than one-thousandth of children had missing values that might be considered acceptable. Third, only limited variables were included in the survey to minimize the time burden to study participants.

## 5. Conclusions

In summary, the exclusive breastfeeding rate under 6 months was quite low and complementary feeding practice was not optimal in China. Complementary feeding indicators varied across residential areas and the rates were lowest in poor rural areas. Low household income and maternal education were potential risk factors for poor complementary feeding practice. Children living in poor rural areas, from poor household or with low maternal education should be given priority for improving complementary feeding, which could be incorporated into the current poverty alleviation program in China.

## Figures and Tables

**Table 1:** General characteristics of the subjects.

Characteristics	*n* (%)
Gender (boy)	51.6% (7464/14,458)
Age group	
0–5 months	30.3% (4381/14,452)
6–11 months	31.8% (4601/14,452)
12–23 months	37.9% (5470/14,452)
Residential area	
Urban-metropolis	22.5% (3251/14,458)
Urban-middle or small cities	28.0% (4045/14,458)
Rural-general areas	32.3% (4677/14,458)
Rural-poor areas	17.2% (2485/14,458)
Ethnicity	
Han	86.6% (12,520/14,453)
Minority	13.4% (1933/14,453)
Maternal Education	
Junior high or below	58.5% (8457/14,456)
Senior high or above	41.5% (5999/14,456)
Maternal age group	
<24 years	20.3% (2930/14,452)
24–26 years	26.1% (3765/14,452)
27–30 years	28.1% (4056/14,452)
>30 years	25.6% (3701/14,452)
Maternal migrant status (Yes)	5.3% (772/14,450)
Annual household income (per capita CNY) *	
≥20,000	27.3% (3940/14,451)
10,000–19,999	28.0% (4039/14,451)
<10,000	35.2% (5082/14,451)

* 1390 of subjects refused to answer the question about annual household income. CNY: Chinese Yuan.

**Table 2:** Determinants of exclusive breastfeeding (EBF) rate for children aged 0–5 months.

Variables	Weighted Percentage of EBF (%)	Crude Odds Ratio (OR) (95% CI: Confidence Interval)	*p*-Value
Gender			0.709
Boy	18.3	0.95 (0.75, 1.22)	
Girl	19.0	1.00	
Duration of children put to the breast after birth			0.116
<1 h	21.4	1.27 (0.94, 1.72)	
≥1 h	17.6	1.00	
Residential area			0.920
Urban-metropolis	18.9	0.87 (0.36, 2.07)	
Urban-middle or small cities	17.5	0.79 (0.36, 1.70)	
Rural-general areas	18.5	0.85 (0.36, 1.98)	
Rural-poor areas	21.2	1.00	
Ethnicity			0.844
Han	18.5	0.94 (0.51, 1.74)	
Minority	19.4	1.00	
Maternal age group			0.945
<24 years	19.5	1.07 (0.70, 1.63)	
24–26 years	18.7	1.01 (0.73, 1.41)	
27–30 years	17.6	0.95 (0.63, 1.42)	
>30 years	18.5	1.00	
Maternal education			0.454
Junior high or below	19.2	1.13 (0.82, 1.54)	
Senior high or above	17.4	1.00	
Maternal migrant status			0.214
Migrant mother	11.0	0.54 (0.20, 1.43)	
Mother living at home	18.8	1.00	
Annual household income * (per capita CNY)			0.550
≥20,000	15.9	0.74 (0.49, 1.12)	
10,000–19,999	18.1	0.87 (0.55, 1.36)	
<10,000	20.3	1.00	

* 1390 of subjects refused to answer the question about annual household income.

**Table 3:** Determinants of minimum dietary diversity (MDD) for children aged 6–23 months.

Variables	Weighted Rate of MDD (%)	Crude OR (95% CI)	Adjusted OR (95% CI)	*p*-Value
Gender ^†^				-
Boy	53.8	1.00 (0.90, 1.12)	-	
Girl	53.7	1.00	-	
Residential area				<0.001
Urban-metropolis	73.3	4.04 (2.42, 6.77)	2.46 (1.36, 4.45)	
Urban-middle or small cities	67.4	3.04 (1.69, 5.46)	2.33 (1.34, 4.05)	
Rural-general areas	43.0	1.11 (0.61, 2.01)	1.05 (0.60, 1.86)	
Rural-poor areas	40.5	1.00	1.00	
Ethnicity ^†^				-
Han	54.2	1.14 (0.62, 2.08)	-	
Minority	50.9	1.00	-	
Maternal age group				0.001
<24 years	44.3	0.59 (0.48, 0.73)	0.73 (0.61, 0.87)	
24–26 years	52.6	0.83 (0.67, 1.02)	0.87 (0.74, 1.03)	
27–30 years	58.8	1.07 (0.91, 1.25)	0.99 (0.83, 1.19)	
>30 years	57.3	1.00	1.00	
Maternal education				<0.001
Junior high or below	46.6	0.39 (0.31, 0.50)	0.61 (0.51, 0.73)	
Senior high or above	69.0	1.00	1.00	
Maternal migrant status				<0.001
Migrant mother	58.5	1.24 (0.91, 1.68)	1.62 (1.26, 2.09)	
Mother living at home	53.3	1.00	1.00	
Annual household income * (per capita CNY)				0.010
≥20,000	66.7	2.58 (1.63, 4.10)	1.63 (1.22, 2.18)	
10,000–19,999	59.0	1.86 (1.30, 2.64)	1.65 (1.13, 2.40)	
<10,000	43.7	1.00	1.00	

^†^ The variable was not included in the multivariate analyses; * 1390 of subjects refused to answer the question about annual household income.

**Table 4:** Determinants of minimum meal frequency (MMF) for children aged 6–23 months.

Variables	Weighted Rate of MMF (%)	Crude OR (95% CI)	Adjusted OR (95% CI)	*p*-Value
Gender ^†^				-
Boy	69.3	1.02 (0.89, 1.16)	-	
Girl	68.9	1.00	-	
Residential area				<0.001
Urban-metropolis	79.7	4.68 (2.68, 8.18)	3.52 (2.06, 5.99)	
Urban-middle or small cities	80.7	5.00 (2.97, 8.43)	4.23 (2.71, 6.60)	
Rural-general areas	66.6	2.38 (1.38, 4.10)	2.27 (1.43, 3.62)	
Rural-poor areas	45.6	1.00	1.00	
Ethnicity				0.600
Han	70.8	1.77 (0.90, 3.47)	1.14 (0.69, 1.88)	
Minority	57.8	1.00	1.00	
Maternal age group				0.767
<24 years	63.0	0.71 (0.55, 0.91)	0.92 (0.73, 1.16)	
24–26 years	69.4	0.94 (0.75, 1.18)	0.99 (0.80, 1.22)	
27–30 years	72.0	1.06 (0.89, 1.27)	1.02 (0.84, 1.24)	
>30 years	70.7	1.00	1.00	
Maternal education				0.001
Junior high or below	64.6	0.49 (0.38, 0.64)	0.73 (0.61, 0.89)	
Senior high or above	78.8	1.00	1.00	
Maternal migrant status ^†^				-
Migrant mother	70.9	1.10 (0.84, 1.44)	-	
Mother living at home	68.9	1.00	-	
Annual household income * (per capita CNY)				0.089
≥20,000	77.0	1.83 (1.14, 2.94)	1.26 (1.00, 1.59)	
10,000–19,999	70.1	1.29 (0.85, 1.97)	1.06 (0.81, 1.40)	
<10,000	64.6	1.00	1.00	

^†^ The variable was not included in the multivariate analyses; * 1390 of subjects refused to answer the question about annual household income.

**Table 5:** Determinants of minimum acceptable diet (MAD) for children aged 6–23 months.

Variables	Weighted Rate of MAD (%)	Crude OR (95% CI)	Adjusted OR (95% CI)	*p*-Value
Gender ^#^				-
Boy	25.5	1.05 (0.95, 1.17)	-	
Girl	24.5	1.00	-	
Residential area				<0.001
Urban-metropolis	44.2	7.53 (4.15, 13.66)	4.59 (2.34, 9.00)	
Urban-middle or small cities	38.1	5.85 (3.04, 11.24)	4.43 (2.26, 8.68)	
Rural-general areas	16.1	1.82 (0.93, 3.57)	1.78 (0.87, 3.66)	
Rural-poor areas	9.5	1.00	1.00	
Ethnicity ^#^				-
Han	25.7	1.30 (0.72, 2.36)	-	
Minority	21.0	1.00	-	
Maternal age group				0.002
<24 years	16.5	0.50 (0.38, 0.65)	0.70 (0.57, 0.85)	
24–26 years	24.5	0.82 (0.66, 1.02)	0.90 (0.76, 1.07)	
27–30 years	29.1	1.04 (0.92, 1.17)	0.97 (0.83, 1.13)	
>30 years	28.4	1.00	1.00	
Maternal education				<0.001
Junior high or below	18.8	0.37 (0.29, 0.48)	0.64 (0.54, 0.76)	
Senior high or above	38.5	1.00	1.00	
Maternal migrant status				0.696
Migrant mother	19.7	0.71 (0.56, 0.90)	0.95 (0.74, 1.22)	
Mother living at home	25.6	1.00	1.00	
Annual household income * (per capita CNY)				<0.001
≥20,000	38.8	2.96 (1.95, 4.48)	1.70 (1.30, 2.23)	
10,000–19,999	26.3	1.66 (1.23, 2.25)	1.39 (1.06, 1.83)	
<10,000	17.6	1.00	1.00	

^#^ The variable was not included in the multivariate analyses; * 1390 of subjects refused to answer the question about annual household income.
